# A Nonparametric Test Reveals Selection for Rapid Flowering in the *Arabidopsis* Genome

**DOI:** 10.1371/journal.pbio.0040137

**Published:** 2006-04-25

**Authors:** Christopher Toomajian, Tina T Hu, Maria José Aranzana, Clare Lister, Chunlao Tang, Honggang Zheng, Keyan Zhao, Peter Calabrese, Caroline Dean, Magnus Nordborg

**Affiliations:** **1**Molecular and Computational Biology, University of Southern California, Los Angeles, California, United States of America; **2**Department of Cell and Developmental Biology, John Innes Centre, Norwich Research Park, Colney, Norwich, United Kingdom; University of EdinburghUnited Kingdom

## Abstract

The detection of footprints of natural selection in genetic polymorphism data is fundamental to understanding the genetic basis of adaptation, and has important implications for human health. The standard approach has been to reject neutrality in favor of selection if the pattern of variation at a candidate locus was significantly different from the predictions of the standard neutral model. The problem is that the standard neutral model assumes more than just neutrality, and it is almost always possible to explain the data using an alternative neutral model with more complex demography. Today's wealth of genomic polymorphism data, however, makes it possible to dispense with models altogether by simply comparing the pattern observed at a candidate locus to the genomic pattern, and rejecting neutrality if the pattern is extreme. Here, we utilize this approach on a truly genomic scale, comparing a candidate locus to thousands of alleles throughout the Arabidopsis thaliana genome. We demonstrate that selection has acted to increase the frequency of early-flowering alleles at the vernalization requirement locus *FRIGIDA.* Selection seems to have occurred during the last several thousand years, possibly in response to the spread of agriculture. We introduce a novel test statistic based on haplotype sharing that embraces the problem of population structure, and so should be widely applicable.

## Abstract 2

A nonparametric approach to detecting selection, based on haplotype sharing throughout the genome, provides strong evidence for selection in *Arabidopsis* at the *FRIGIDA* locus responsible for early flowering.

## Introduction

**Figure 1:**
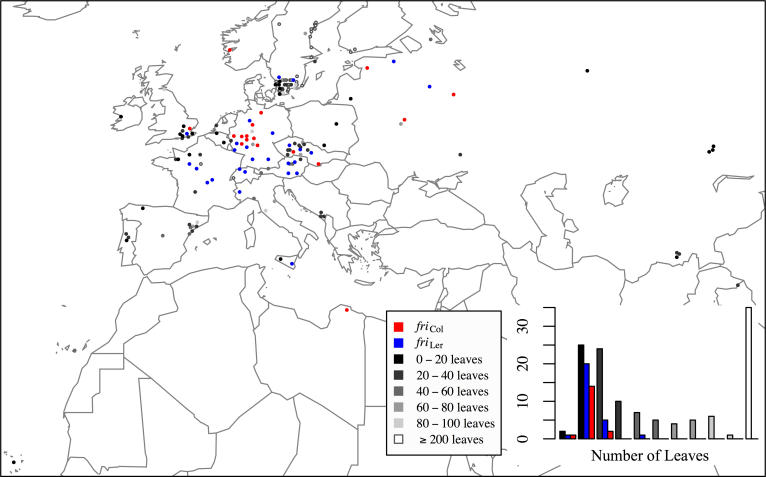
The Effect of Geography and *FRI* Alleles on Flowering Time The map displays the position of 168 accessions from Europe, northern Africa, and western Asia. The histogram inset shows the distribution of the number of leaves at flowering without vernalization for these accessions. In both map and histogram, accessions carrying the *fri _Col_* or *fri _Ler_* alleles are indicated in red or blue, respectively, while the shade of gray in the remaining accessions corresponds to the number of leaves (i.e., 0–20). Bin sizes are halved (i.e., 0–10 leaves) in the histogram relative to the map.

The coordination of flowering time with environmental factors is a major determinant of reproductive success in plants, and numerous experiments in many different species have shown that populations are typically strongly adapted to their local environment. Plants decide when to flower by integrating multiple environmental and endogenous inputs: the underlying pathways are best understood in *Arabidopsis thaliana,* which has become the major model for studying flowering time [ 1]. A. thaliana occurs throughout the Northern Hemisphere in a wide range of environments, and shows considerable variation for flowering time [ 2– 10]. In particular, many genotypes are extremely late-flowering unless exposed to prolonged cold temperatures—so-called vernalization—which likely functions to ensure a winter-annual habit. Several studies have shown that the locus *FRIGIDA (FRI)* explains much of this variation [ 5– 10]. Recessive loss-of-function alleles at this locus essentially eliminate the requirement for vernalization [ 11]. Two such alleles, *fri _Col_* and *fri _Ler_* (originally defined in the common laboratory accessions Columbia [Col] and Landsberg *erecta* [Ler]), account for a large proportion of early-flowering accessions found in Europe ( Figure 1). Given their strong effect on a trait likely to be under strong selection, there is every reason to believe that these alleles have been under selection. Consistent with this, a study of polymorphism in the *FRI* chromosomal region suggested that they were both associated with long haplotypes (i.e., for each allele, most or all individuals carrying that allele also shared identical haplotypes over long chromosomal regions), possibly as a result of selection [ 12]. However, without a genomic control, there was no way of determining whether the length of these haplotypes was indeed unusual. Here, we evaluate the evidence for recent selective sweeps on *fri _Col_* and *fri _Ler_* by investigating patterns of haplotype sharing throughout the genome. Formally, we seek to reject the null hypothesis that the two loss-of-function alleles do not exhibit extremely high haplotype sharing (as would be expected if they had not been affected by recent selection), in favor of the alternative hypothesis that they do [ 13]. We use polymorphism data from 96 individual specimens of A. thaliana for which 1,102 short fragments have been sequenced as part of an ongoing survey of genomic polymorphism [ 14]. The density of our sequenced regions along each chromosome is sufficient to identify unusually long identical haplotypes among subsets of these individuals.

The rationale behind our approach is that chromosomes that are identical by descent at a polymorphic site must also share a short region surrounding that site. The length of this identical by descent region is influenced primarily by the age of the shared allele at the polymorphic site and the recombination rate in the region [ 15, 16]. A mutation that is driven by directional selection will typically have reached its current population frequency much faster than a mutation that reached the same frequency as a result of genetic drift alone [ 17]. This leaves less time for recombination and mutation to break up the ancestral haplotype, resulting in a larger region of identity by descent around the mutation than expected given its current frequency [ 18– 21]. Several studies have suggested methods for detecting this signature of recent selection [ 22– 24]. A limitation common to all is that they rely on population genetics models to determine significance, models that are based on numerous assumptions about the demography and geographic structure of the population. In contrast, we take a nonparametric approach to detecting selection, and determine significance by comparing haplotype sharing around *FRI* with what is observed in the rest of the genome [ 25, 26]. Our analysis shows that haplotype sharing around the two major *FRI* loss-of-function alleles is indeed unusually high, thus providing strong evidence for selection on these alleles.

## Results/Discussion

In order to compare haplotype sharing around the loss-of-function alleles at *FRI* with sharing at thousands of reference loci throughout the genome, we developed a novel haplotype-sharing measure. In particular, we tried to develop a measure that accounts for population structure, because our sample is heavily structured [ 14]. Thus, pairs of individuals from the same population are more likely to share long haplotypes because they are more closely related than those from different populations. For a formal definition of our pairwise haplotype-sharing score (PHS), and details on how we calculate it, see Materials and Methods. Informally, the basis of this measure is the estimation of the shared length around any allele *A* at position *x* from a simple pairwise comparison between individual haplotypes [ 27]. Thus, haplotype sharing relative to allele *A* can only be measured if this allele is found on at least two haplotypes. Note that, due to inbreeding, comparisons can be made directly between individuals (i.e., haplotypes need not be inferred from genotypes). To account for population structure and increase our power to detect true footprints of selection, we normalized the length of identity of a pair of individuals around each allele by subtracting the mean length of identity found in these two individuals (across all polymorphic loci) and dividing by the standard deviation. Then, we averaged these normalized lengths over all pairs of individuals carrying allele *A.* A final concern is that regions of the genome where we erroneously infer high haplotype sharing due to the low density of our sequenced regions may have incorrectly inflated PHS scores. In order to guard against this possibility, the final step in calculating PHS is the subtraction of the normalized lengths around position *x* averaged over all pairs of individuals, regardless of which allele they carry. As a result, our measure misses a selective sweep that has affected the whole sample, and has reduced power to detect a sweep that has affected most of it: it is geared towards identifying alleles that are present in a minority of individuals.

### Haplotype Sharing around *FRI* Is Extreme

**Figure 2:**
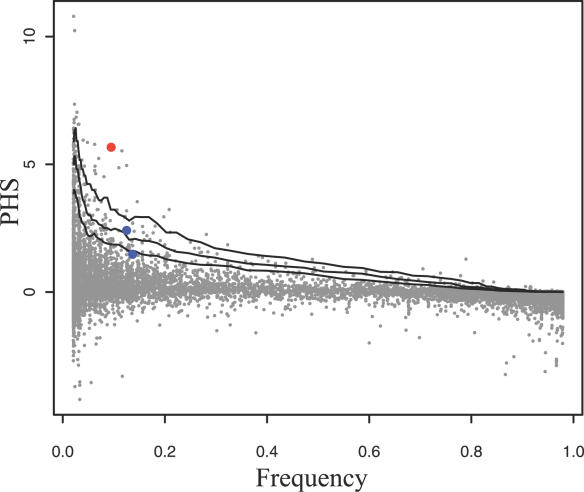
PHS Plotted against Frequency for all Nonredundant Alleles in Our Genome-Wide Dataset (10,961 Alleles) The percentile lines (95th, 97.5th, and 99th) are computed from the respective percentiles (and average frequency) of 100 overlapping sliding windows (100-point offset between adjacent windows) of 1,000 points along the data (sorted by frequency). The *fri _Col_* allele is shown in red, while the *fri _Ler_* allele and another allele that marks it are shown in blue. *fri _Ler_* is the allele below the 95th percentile line.

We calculated PHS for all alleles in our data set where, at the corresponding polymorphic site, the minor allele is found in at least two accessions and we have data from at least 60 accessions. We also calculated the measure for *fri _Col_* and *fri _Ler_,* which have been genotyped in our sample. The distribution of this measure, plotted against the frequency of each allele, is shown in Figure 2. Often, nearby alleles are highly correlated and provide redundant information about a long shared haplotype. Therefore, we eliminated all but the highest-scoring allele from sets of nearby correlated alleles to reduce this redundancy (see Materials and Methods). The average value of the haplotype-sharing measure decreased with increasing allele frequency as expected. In order to help identify abnormally high values for any given frequency, we plotted lines representing the 95th, 97.5th, and 99th percentile for sliding windows of increasing frequency. The *fri _Col_* allele is well above the 99th percentile line, providing strong support for a recent selective sweep. Formally, our test rejects the null hypothesis that this allele is consistent with the vast majority of (presumably neutral) alleles in the genome at the 0.01 level. The *fri _Ler_* allele also shows extensive haplotype sharing but considerably less so than *fri _Col_.* A tightly linked allele shared by 12 of the 13 accessions that carry *fri _Ler_* lies just above the 97.5th percentile line. Because this allele has a higher PHS score than *fri _Ler_,* the latter is formally eliminated from our analysis. The *fri _Ler_* allele itself is included in Figure 2 for the sake of comparison: it lies just below the 95th percentile line.

An implicit assumption in our test that compares two candidate alleles against thousands of alleles throughout the genome, which serve as reference alleles, is that these reference alleles are selectively neutral. This is almost certainly false. However, violation of this assumption in this and related tests should make the test conservative. Examples of recent selection among reference loci will shift the genome-wide distribution of PHS to higher values, making it less likely that an individual candidate locus will reach statistical significance. Only a selective force that systematically decreased PHS values in a sizable number of reference loci would result in inflated *p* values in tests of individual candidate loci. To our knowledge, the only force that might be capable of this is rampant long-term balancing selection maintaining ancient polymorphism (with ancient polymorphism producing low PHS scores, just as young alleles tend to produce high ones). Leaving aside the biological plausibility of this explanation (especially in a highly selfing organism, where heterozygote advantage is unlikely to maintain variation), we do not believe it would have the required effect on the PHS scores. Specifically, due to a number of factors, in particular the density of our polymorphism data and the conservative approach taken to estimate haplotype sharing, we do not believe extremely low values of PHS accurately reflect unusually short haplotype sharing. Thus, we feel that long-term balancing selection has a negligible effect on the distribution of PHS in our reference loci, and we also do not believe that low PHS scores can be used here to identify ancient alleles preserved by long-term balancing selection. It is possible that a haplotype-sharing measure such as ours would perform better with higher-density polymorphism data, but we are skeptical that such an approach would be more powerful than those that use polymorphism-to-divergence ratios. The issue deserves further study.

### EHH and the Effect of Population Structure

We also calculated haplotype sharing using a modification of the extended haplotype homozygosity (EHH) measure [ 23, 28], which makes no correction for population structure. Briefly, for each allele where we have calculated PHS, we calculated EHH at flanking markers of increasing distance. We interpolated the distance at which EHH drops below 0.5 and used this as a score. We similarly removed all but the highest scoring alleles from nearby highly correlated alleles. The distribution of this score against allele frequency is superficially similar to that of PHS (not shown). Again, the *fri _Col_* allele is above the 99th percentile given its frequency, and the *fri _Ler_* allele is just above the 95th percentile.

In general, however, alleles that exhibit extreme haplotype sharing using PHS do not necessarily do so using EHH (and vice versa). Because the PHS measure makes adjustments for some pairs of individuals being more related than others (see Materials and Methods), while EHH does not, we would expect the latter measure to sometimes identify haplotype sharing that can be attributed to population structure rather than selection. To investigate this, we compared alleles with extreme values with respect to which individuals carried them. Separately for the PHS and EHH analyses, we clustered all alleles above the 99th percentile using unweighted pair group method with arithmetic averages (UPGMA) and a distance metric that measures whether alleles tend to be carried by the same individuals or not. The distance metric we used, the binary distance, treats each allele as a vector of binary bits (corresponding to the accessions), where the value of each bit (0 or 1) indicates whether the corresponding accession carries the given allele. The distance between two alleles is then the number of bits for which one allele is 0 and the other 1, divided by the total number of bits with at least one 1. To reduce noise, we restricted the analysis to alleles found in more than two individuals, and at frequency less than 0.5. If population structure were responsible for haplotype sharing, we would expect the same individuals to share haplotypes at several loci and the relevant alleles would be clustered. Indeed, in the EHH analysis, we found seven sets of low-frequency alleles that clustered with an average distance less than or equal to 0.5. Three pairs of alleles and one group of five alleles clustered with an average distance of less than 0.4 ( Figure S1). In one extreme case, two alleles were found in exactly the same set of accessions. The clustered alleles tend to be found in restricted geographic regions. For example, five alleles found exclusively in the United States cluster together, and four pairs and one triplet of alleles found predominantly in northern Sweden cluster together. This is consistent with the high haplotype sharing of these alleles resulting solely from the population structure present in the sample. In contrast, only three clusters of low-frequency alleles resulted from the PHS analysis, and just a single pair of alleles clustered at a distance of less than 0.4. These results demonstrate that, as expected, the EHH analysis is much more susceptible to identifying multiple alleles from the same sets of closely related individuals than the PHS measure when population structure is present in the sample. However, for organisms with a less extensive population structure than *A. thaliana,* such as humans, we expect the methods will yield very similar results.

### The History of the FRI Alleles

What does the pattern of haplotype sharing and linkage disequilibrium (LD) around *fri _Col_* and *fri _Ler_* tell us about the history of these alleles? Age estimates can be obtained by counting the proportion of chromosomes carrying the allele that retain the inferred ancestral haplotype at linked markers. Following the approach of Stephens et al. [ 29], and treating fragments flanking *FRI* as multiallelic markers, we obtained rough age estimates of approximately 800 generations for *fri _Col_* and 3,200 generations for *fri _Ler_.* The greater estimated age of *fri _Ler_* reflects the fact that haplotype sharing among these alleles is less extreme than for *fri _Col_* and is consistent with the greater geographic spread of the former allele ( Figure 1). More exact estimates are not possible without better estimates of the rates of outcrossing and recombination, and more information on population structure in *A. thaliana,* all of which affect the decay of LD. Our age estimates are inversely proportional to the rates of recombination in this region and historical outcrossing in this species. Therefore, if we assume we have overestimated either of these rates by a factor of two and recalculate age after halving the inflated rate, the ensuing age estimates would double. Nevertheless, assuming at least one generation per year for these rapid-cyclers, our estimates suggest that selection for null alleles of *FRI* may have occurred more recently than the colonization of northern Europe by *A. thaliana,* which necessarily followed the last glacial retreat estimated at approximately 13,000 years ago [ 30].

It is tempting to speculate that these alleles, the effect of which is to convert an obligate winter-annual plant into one that can have multiple generations per year, have spread as a result of selection for weediness imposed by agriculture. Two other observations support the notion that the *FRI*-null alleles represent an adaptation to some form of human disturbance: first, flowering time variation in A. thaliana seems to be much more strongly correlated with geography (in particular latitude) if individuals carrying *FRI*-null alleles are excluded than if they are not excluded [ 8]; second, while A. thaliana accessions in general show strong isolation by distance, accessions carrying *fri _Col_* found far from Germany (the center of distribution of this allele) generally are genetically similar to other German accessions [ 14], suggesting recent dispersal. More research is required to clarify the exact mechanism of selection.

### Scanning the Genome for Recent Selection

Thus far we have demonstrated how the genomic distribution of haplotype sharing may be used in a priori tests of selection when strong candidates, such as the *FRI* loss-of-function alleles, exist. We now turn to a fundamentally different application, namely scanning the genome for signs of recent selection. Because we have no a priori candidates, we are simply trying to identify those alleles and regions that exhibit the strongest signals of selection [ 31, 32]. However, since we are not assuming any statistical model, we have no way of assessing the significance of our findings. The top 1% is just that. Nonetheless, assuming that some recent selection has occurred in the genome, we expect that this set of alleles and their associated haplotypes will be enriched for regions affected by recent selection, and thus each of these regions can be considered as a candidate for recent selection.

In addition to *fri _Col_,* 31 alleles found in more than two accessions and at frequency less than 0.8 were identified as clearly above the 99th percentile of PHS ( Table S1). They were found on all five chromosomes, though they were not distributed evenly according to genetic length (χ ^2^ 4 degrees of freedom, *p* < 0.005), with an excess found on Chromosomes 2 and 4. Some of these alleles, hereafter referred to as candidate alleles, may be associated with targets of selection; most are probably due to chance. We certainly plan to investigate a few of the more interesting regions further.

A problem when using haplotype sharing to scan the genome for selection is that it is difficult to identify the precise target of selection. Given that our polymorphism data represent approximately 0.5% of the genome, it is likely that the targets of selection reside in the 99.5% of the genome between our sequenced fragments and that most of the 31 candidate alleles merely are in strong LD with them. But even if we had complete sequence information, it would be difficult to identify the targets of selection. This is because selection at a single allele will often generate a complex pattern of increased haplotype sharing around multiple alleles throughout the chromosomal region. We attempted to remove alleles from our analyses that were obviously part of the same event. For instance, alleles in two fragments linked to *FRI* that were in strong LD with the *fri _Col_* allele had PHS scores that are above the 99 ^th^ percentile, but because these alleles were redundant with *fri _Col_* and scored lower, they were removed. Yet our criteria for identifying redundant alleles are arbitrary, and will not always work.

**Figure 3:**
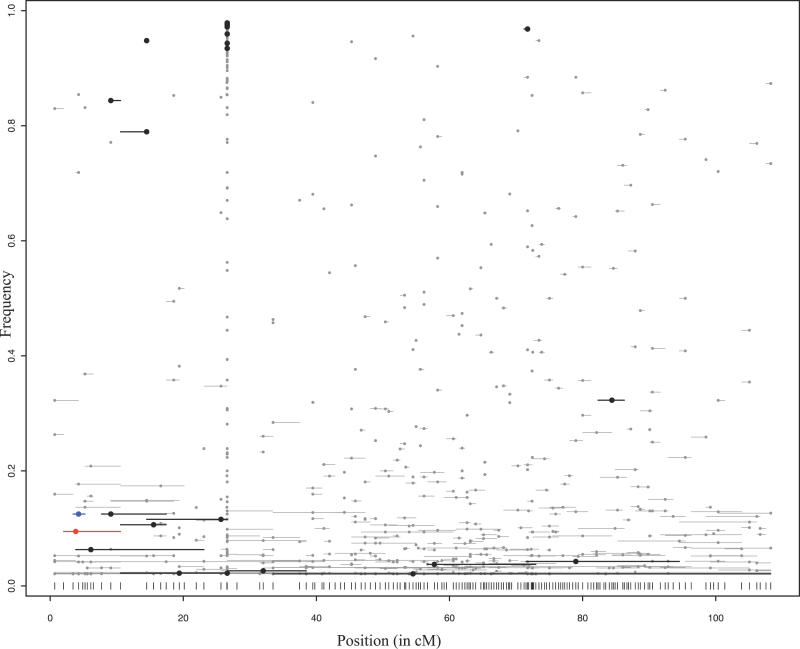
Haplotype Sharing of Alleles Plotted by Frequency along Chromosome 4 Points represent the location of alleles, while corresponding lines indicate the average length of haplotype identity flanking the allele. Those alleles present in the upper 99th percentile are indicated in black. The *fri _Col_* allele and associated haplotype are indicated in red. This haplotype is over 600 kb, or 8 cM, long. The allele marking *fri _Ler_* and its associated haplotype are indicated in blue. Only those alleles with a positive score are plotted. Locations of the sequence fragments are indicated at the bottom of the figure.

Our analysis identified two regions with complex patterns of haplotype sharing, such as those detailed above, involving at least five candidate alleles within a single Mb. The most extreme example is Chromosome 2, where nine candidate alleles were found between positions 9.2 and 11.6 Mb. The other example is the short arm of Chromosome 4, where five candidate alleles were found between 0.2 and 1.2 Mb and a sixth was just short of the centromere at 2.4 Mb. The *FRI* gene is located at 0.2 Mb, and it is illuminating to consider the role of *FRI* in the overall pattern of haplotype sharing in this region. In Figure 3, we have plotted the location and extent of regions of haplotype sharing along Chromosome 4 (for the remaining chromosomes, see Figures S2– S5). On the short arm, four candidate alleles were minor alleles, and two of these, found at 1.1 Mb and 2.4 Mb, shared more than a single accession in common with *fri _Col_.* The allele at 2.4 Mb is associated with a haplotype that encompasses a reported pericentric inversion between the Columbia and Landsberg accessions [ 33]. This inversion, located at the opposite end of the chromosome arm from *FRI,* is expected to interfere with recombination between two haplotype classes, and may result in errors in our estimates of both distance and haplotype sharing in the immediate region. Although the *fri _Col_* allele was carried by two individuals that have no mismatches in our data through the inversion, the remaining individuals also displayed high levels of haplotype sharing, indicating that the haplotype sharing around *fri _Col_* cannot be explained by the inversion. For *fri _Col_* and the alleles at 1.1 Mb and 2.4 Mb, we removed the accessions that carried each allele in turn, and recalculated PHS for the other two alleles. In each case, the remaining two alleles were still extreme given their observed frequency, indicating that haplotype sharing around each allele cannot be explained by the other alleles. The remaining minor frequency alleles appeared to be independent of *fri _Col_,* but it is impossible to rule out selection on this allele having induced extensive haplotype sharing among other accessions. The final candidate allele in this region, a major frequency allele, was too common to test its independence from other alleles in this region by removing all accessions that carry it. A detailed view of haplotype structure along the short arm of Chromosome 4 is provided in Figure S6.

### Concluding Remarks

Taken together, the effect, frequency, and geographic distribution of the two most common *FRI* loss-of-function alleles had strongly suggested that they had been involved in adaptation. Our analysis provides strong support for this conjecture for at least one of these alleles, *fri _Col_.* The evidence for selection on the other allele, *fri _Ler_,* is weaker: given that the two alleles have very similar phenotypic effects, the simplest explanation for this is that the selective sweep took place longer ago. Our genome-wide scan for selection has also identified many other regions that are candidates for harboring genes that have been the subject of recent selective sweeps. As we learn more about the genes responsible for natural variation in this organism, it will be interesting to see how many lie in these putatively selected chromosomal regions. The type of scan developed here should be useful in a wide range of species with cryptic population structure, and with some modification for genotype data, could be applied to data collected by the Human International Haplotype Mapping Project.

## Materials and Methods

### Genome-wide polymorphism dataset and sample choice

The majority of data used here has already been described [ 14], and the rest were gathered in the same manner. Briefly, we have sequenced 1,102 fragments of approximately 500 bp in a set of 96 accessions. The mean and median distance between adjacent fragments are 107.5 and 96.6 kb, respectively, or an estimated 0.49 and 0.40 cM, respectively. Full alignments for these fragments are provided in Dataset S1. The 96 accessions were chosen for the previous study both to capture some information on within-region variation in *Arabidopsis* as well as include accessions that have been commonly used in the creation of recombinant inbred lines. The fact that we account for population structure in our approach implies that our results should be robust to sample composition. We took the sampled frequency of each allele as an estimate of its global frequency. As the PHS score shows a decline with increasing frequency, errors in these global frequency estimates could affect our results. However, accessions were not chosen on the basis of *FRI* genotypes.

### PHS

We defined the haplotype-sharing statistic



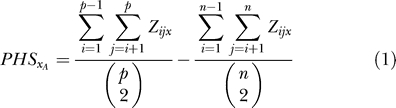



where *p* is the number of individuals carrying allele *A* at position *x,* and *n* is the sample size at position *x;* and



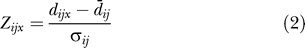



where *d _ijx_* is the distance on a genetic map over which individuals *i* and *j* are identical around position *x,* *d¯ _ij_* is the mean of the genome-wide distribution of distances over which these individuals are identical, *σ _ij_* is the standard deviation of that distribution, and individuals *i* and *j* share the same allele at position *x.* The normalized genetic distance *Z _ijx_* was employed in order to control for population structure in our data. The final statistic is expressed as the difference of two averages (the first, averaged over all pairs that share the allele; the second, over all possible pairs) to guard against genome regions where on average all pairs of accessions are identical for long stretches, either from few sequenced fragments, or few or low-frequency polymorphisms in the available sequences.

Heterozygous sites (rare in our data) were converted to missing data. Missing data values do not interrupt haplotype identity between pairs of individuals, nor do they count toward extending identical haplotypes until they are followed by a polymorphism where both individuals are identical.

For each allele we also calculated the average genetic distance to the left and right that any two individuals carrying the allele are identical and recorded these as the endpoints of sharing for that allele. Because our polymorphism data represents only a fraction of the whole genome, we can usually only infer the location where identical haplotypes first differ. To be conservative, we measured haplotype identity out to the last whole adjacent fragment that is identical between the pair of individuals. These endpoints of haplotype sharing are approximate and serve only as a guide to the extent of individual regions of haplotype sharing.

### Filtering highly correlated alleles

LD between nearby alleles is often very high. Because we exhaustively calculate PHS for each allele in our dataset for all polymorphisms that have genotypes from at least 60 individuals, this often leads to the same score for many nearby alleles that are perfectly correlated. In this case, each of these alleles represents the same block of haplotype sharing. To reduce over-counting blocks of haplotype sharing, we removed all but the highest scoring allele from each group of correlated alleles. Correlated alleles are those between which *r* ^2^ > 0.5 and *r* is positive, and they are within the same fragment or their intervals of haplotype sharing overlap. We expect this filtering reduces the amount of redundancy in the high scoring alleles, though it is likely that pairs or groups of alleles still remain that are extreme due to the same shared haplotypes.

### EHH analysis

As EHH for a given allele or haplotype can be calculated at many flanking markers, it is not obvious how to compare this measure with PHS, which has a unique value. To make PHS and EHH more comparable, we calculated a score based on EHH as follows. For each allele where we have calculated PHS, we also calculated EHH (and EHH for all individuals who do not carry the given allele) at flanking polymorphic sites increasingly distant from the allele. Independently to the left and right, we found the polymorphic sites between which EHH drops below 0.5 and interpolated the position where this occurs. The score is then the total distance on a genetic map between the two positions thus estimated [ 28]. The score was assigned to zero for all alleles where this genetic distance is less than or equal to the genetic distance between the sites where EHH measured for all individuals who do not carry the allele drops below 0.5.

### Estimates of distances on a genetic map

Functions to translate physical distances into distances on a genetic map (i.e., recombination frequencies) were created for each chromosome. Briefly, a third-order polynomial was fitted to data on position on physical and genetic maps for markers on each chromosome arm. We used a total of 483 markers from http://www.arabidopsis.org, where positions on both physical and genetic maps are available. In Supporting Information we include Table S2 describing these markers, as well as Figures S7– S11, which illustrate the fitted curves of genetic by physical distance for each chromosome and the markers used to produce those curves. For the first arm of each chromosome, the polynomial was forced through the origin. In the physical region between chromosome arms representing each centromere, an estimate of the genetic position of the centromere was applied. The regions that approximated the physical extent of each centromere were given a zero genetic distance. In order that the function may be monotonically increasing, a short linear segment was added between the first arm of Chromosome 4 and its centromere, where the fitted polynomial started to decrease.

### Age estimates of alleles

Following Equation 2 of Stephens et al. [ 29], we obtained estimates of the time to the most recent common ancestor of the *fri _Col_* and *fri _Ler_* alleles from the breakdown of LD at flanking fragments. We assigned the ancestral haplotype of each *FRI* allele at each fragment as the majority haplotype among the individuals that were identical from the core allele to the previous fragment. In estimating *r,* the rate of change from the ancestral haplotype at each fragment, we used the genetic distance from *FRI* (a proxy for recombination fraction) multiplied by the proportion of recombination events resulting from outcrossing that would result in the loss of the ancestral haplotype (i.e., the proportion of chromosomes not carrying the specified deletion that do not carry the deletion's ancestral haplotype) and the rate of outcrossing, here taken to be 0.01. Uncertainty exists in the rates of both recombination and outcrossing, and this uncertainty is difficult to quantify [ 34], though present day outcrossing estimates from disturbed populations may greatly overestimate historical outcrossing rates. These rates are linearly proportional to *r,* which is inversely proportional to the estimated time, meaning overestimates of them lead directly to underestimates of time. For *fri _Col_* and *fri _Ler_,* we reported the mean time to the most recent common ancestor from estimates at seven and nine flanking fragments, respectively.

## Supporting Information

Dataset S1 All Data Used in This Paper, Including Genome-Wide Sequence Alignments, *FRI* Genotypes, and Flowering Time Data
(1.79 MB ZIP)

Figure S1 Clustering of High-Scoring Alleles from PHS and EHH Analyses Clustering is based on the similarity among alleles of which individuals carry them (measured by binary distance). Clustering of alleles from the EHH analysis is presented at the top of the figure, while PHS clustering is below. Colored boxes highlight clusters where the mean binary distance between alleles is less than or equal to 0.5. To the right of the clusters are color-coded labels describing the regions where the boxed alleles predominate.
(28 KB PDF)

Figure S2 Haplotype Sharing of Alleles Plotted by Frequency along Chromosome 1 (11.6 MB PDF)

Figure S3 Haplotype Sharing of Alleles Plotted by Frequency along Chromosome 2 (146 KB PDF)

Figure S4 Haplotype Sharing of Alleles Plotted by Frequency along Chromosome 3 (5.6 MB PDF)

Figure S5 Haplotype Sharing of Alleles Plotted by Frequency along Chromosome 5 (6.4 MB PDF)

Figure S6 Haplotype Structure of the Short Arm of Chromosome 4 For each alignment in this region, nonredundant (i.e., *r ^2^* = 1) polymorphisms are indicated in columns and accessions in rows. Minor frequency alleles are black and major frequency alleles are gray, while missing data or spaces between alignments are white. A genetic scale (in cM) is included at the top of the figure, and the position of each alignment on it is indicated. The extended haplotypes surrounding seven alleles that are in the upper 2.5% tail of genome-wide PHS and that are below 15% frequency are boxed, and the fragment where the core allele is located is also boxed. The haplotype surrounding *fri _Col_* is boxed in red, and that surrounding the marker allele for *fri _Ler_* in blue. Due to topological constraints, one accession belonging to the third group from the top is not shown in this group because it is included with the *fri _Ler_* marker group.
(5.1 MB JPG)

Figure S7 Fitted Curves Relating Genetic and Physical Positions for Chromosome 1 Lines represent third-order polynomials fitted to markers (points) for each chromosome arm and the centromere. The density of sequence alignments is indicated along both axes.
(73 KB PDF)

Figure S8 Fitted Curves Relating Genetic and Physical Positions for Chromosome 2 (56 KB PDF)

Figure S9 Fitted Curves Relating Genetic and Physical Positions for Chromosome 3 (61 KB PDF)

Figure S10 Fitted Curves Relating Genetic and Physical Positions for Chromosome 4 (58 KB PDF)

Figure S11 Fitted Curves Relating Genetic and Physical Positions for Chromosome 5 (63 KB PDF)

Table S1 Description of Alleles Associated with High Haplotype Sharing (22 KB XLS)

Table S2 List of Genetic Markers Used (91 KB XLS)

### Accession Numbers

The Entrez Gene ( http://www.ncbi.nlm.nih.gov/gquery/gquery.fcgi) accession number for *FRI* is 828044.

## Abbreviations

Col: Columbia
EHH: extended haplotype homozygosity
*FRI*: *FRIGIDA*
*LD*: linkage disequilibrium
Ler: Landsberg *erecta*
PHS: pairwise haplotype sharing

## References

[pbio-0040137-b001] Simpson GG, Dean C (2002). *Arabidopsis*, the rosetta stone of flowering time?. Science.

[pbio-0040137-b002] Karlsson BH, Sills GR, Nienhuis J (1993). Effects of photoperiod and vernalization on the number of leaves at flowering in 32
*Arabidopsis thaliana (Brassicaceae)* ecotypes. Am J Bot.

[pbio-0040137-b003] Sanda SL, John M, Amasino RM (1997). Analysis of flowering time in ecotypes of
Arabidopsis thaliana. J Hered.

[pbio-0040137-b004] Nordborg M, Bergelson J (1999). The effect of seed and rosette cold treatment on germination and flowering time in some
*Arabidopsis thaliana (Brassicaceae)* ecotypes. Am J Bot.

[pbio-0040137-b005] Gazzani S, Gendall AR, Lister C, Dean C (2003). Analysis of the molecular basis of flowering time variation in
*Arabidopsis* accessions. Plant Physiol.

[pbio-0040137-b006] Michaels SD, He YH, Scortecci KC, Amasino RM (2003). Attenuation of
*FLOWERING LOCUS C* activity as a mechanism for the evolution of summer-annual flowering behavior in
*Arabidopsis*. Proc Natl Acad Sci USA.

[pbio-0040137-b007] Hagenblad J, Tang C, Molitor J, Werner J, Zhao K (2004). Haplotype structure and phenotypic associations in the chromosomal regions surrounding two
Arabidopsis thaliana flowering time loci. Genetics.

[pbio-0040137-b008] Stinchcombe JR, Weinig C, Ungerer M, Olsen KM, Mays C (2004). A latitudinal cline in flowering time in
Arabidopsis thaliana modulated by the flowering time gene
*FRIGIDA*. Proc Natl Acad Sci USA.

[pbio-0040137-b009] Shindo C, Aranzana MJ, Lister C, Baxter C, Nicholls C (2005). Role of
*FRIGIDA* and
*FLOWERING LOCUS C* in determining variation in flowering time of
*Arabidopsis*. Plant Physiol.

[pbio-0040137-b010] Werner JD, Borevitz JO, Uhlenhaut NH, Ecker JR, Chory J (2005). *FRIGIDA*-independent variation in flowering time of natural
A. thaliana accessions. Genetics.

[pbio-0040137-b011] Johanson U, West J, Lister C, Michaels S, Amasino R (2000). Molecular analysis of
*FRIGIDA*, a major determinant of natural variation in
*Arabidopsis* flowering time. Science.

[pbio-0040137-b012] Hagenblad J, Nordborg M (2002). Sequence variation and haplotype structure surrounding the flowering time locus
*FRI* in
Arabidopsis thaliana. Genetics.

[pbio-0040137-b013] Taylor MFJ, Shen Y, Kreitman ME (1995). A population genetic test of selection at the molecular-level. Science.

[pbio-0040137-b014] Nordborg M, Hu TT, Ishino Y, Jhaveri J, Toomajian C (2005). The pattern of polymorphism in
Arabidopsis thaliana. PLoS Biol.

[pbio-0040137-b015] Nordborg M, Tavaré S (2002). Linkage disequilibrium: What history has to tell us. Trends Genet.

[pbio-0040137-b016] Innan H, Nordborg M (2003). The extent of linkage disequilibrium and haplotype sharing around a polymorphic site. Genetics.

[pbio-0040137-b017] Maruyama T (1974). The age of an allele in a finite population. Genet Res.

[pbio-0040137-b018] Maynard Smith J, Haigh J (1974). The hitch-hiking effect of a favourable gene. Genet Res.

[pbio-0040137-b019] Kaplan NL, Hudson RR, Langley CH (1989). The “hitchhiking effect” revisited. Genetics.

[pbio-0040137-b020] Hudson RR, Bailey K, Skarecky D, Kwiatowski J, Ayala FJ (1994). Evidence for positive selection in the superoxide-dismutase (SOD) region
*of Drosophila melanogaster*. Genetics.

[pbio-0040137-b021] Kim Y, Stephan W (2002). Detecting a local signature of genetic hitchhiking along a recombining chromosome. Genetics.

[pbio-0040137-b022] Slatkin M, Bertorelle G (2001). The use of intraallelic variability for testing neutrality and estimating population growth rate. Genetics.

[pbio-0040137-b023] Sabeti PC, Reich DE, Higgins JM, Levine HZ, Richter DJ (2002). Detecting recent positive selection in the human genome from haplotype structure. Nature.

[pbio-0040137-b024] Toomajian C, Ajioka RS, Jorde LB, Kushner JP, Kreitman M (2003). A method for detecting recent selection in the human genome from allele age estimates. Genetics.

[pbio-0040137-b025] Kroymann J, Mitchell-Olds T (2005). Epistasis and balanced polymorphism influencing complex trait variation. Nature.

[pbio-0040137-b026] Yu F, Sabeti PC, Hardenbol P, Fu Q, Fry B (2005). Positive selection of a pre-expansion CAG repeat of the human
*SCA2* gene. PLoS Genet.

[pbio-0040137-b027] Mathews DJ, Kashuk C, Brightwell G, Eichler EE, Chakravarti A (2001). Sequence variation within the
*Fragile X* locus. Genome Res.

[pbio-0040137-b028] Sabeti PC, Walsh E, Schaffner SF, Varilly P, Fry B (2005). The case for selection at CCR5-Δ32. PLoS Biol.

[pbio-0040137-b029] Stephens JC, Reich DE, Goldstein DB, Shin HD, Smith MW (1998). Dating the origin of the CCR5-Δ32 AIDS-resistance allele by the coalescence of haplotypes. Am J Hum Genet.

[pbio-0040137-b030] Hewitt GM (2001). Speciation, hybrid zones and phylogeography—or seeing genes in space and time. Mol Ecol.

[pbio-0040137-b031] Akey JM, Zhang G, Zhang K, Jin L, Shriver MD (2002). Interrogating a high-density SNP map for signatures of natural selection. Genome Res.

[pbio-0040137-b032] The International HapMap Consortium (2005). A haplotype map of the human genome. Nature.

[pbio-0040137-b033] Fransz PF, Armstrong S, de Jong JH, Parnell LD, van Drunen C (2000). Integrated cytogenetic map of Chromosome arm 4S in
*A. thaliana* Structural organization of heterochromatic knob and centromere region. Cell.

[pbio-0040137-b034] Abbott RJ, Gomes MF (1989). Population genetic structure and outcrossing rate of
Arabidopsis thaliana (L.) Heynh. Heredity.

