# Supplementary material for: A Nonparametric Test Reveals Selection for Rapid Flowering in the Arabidopsis Genome
Source: PLoS Biol. 2006 Apr 25;4(5):e137. doi: 10.1371/journal.pbio.0040137 (PMC1440937; doi:10.1371/journal.pbio.0040137)
Supplement: Supplementary file 1 [file pbio.0040137.sg001.pdf]

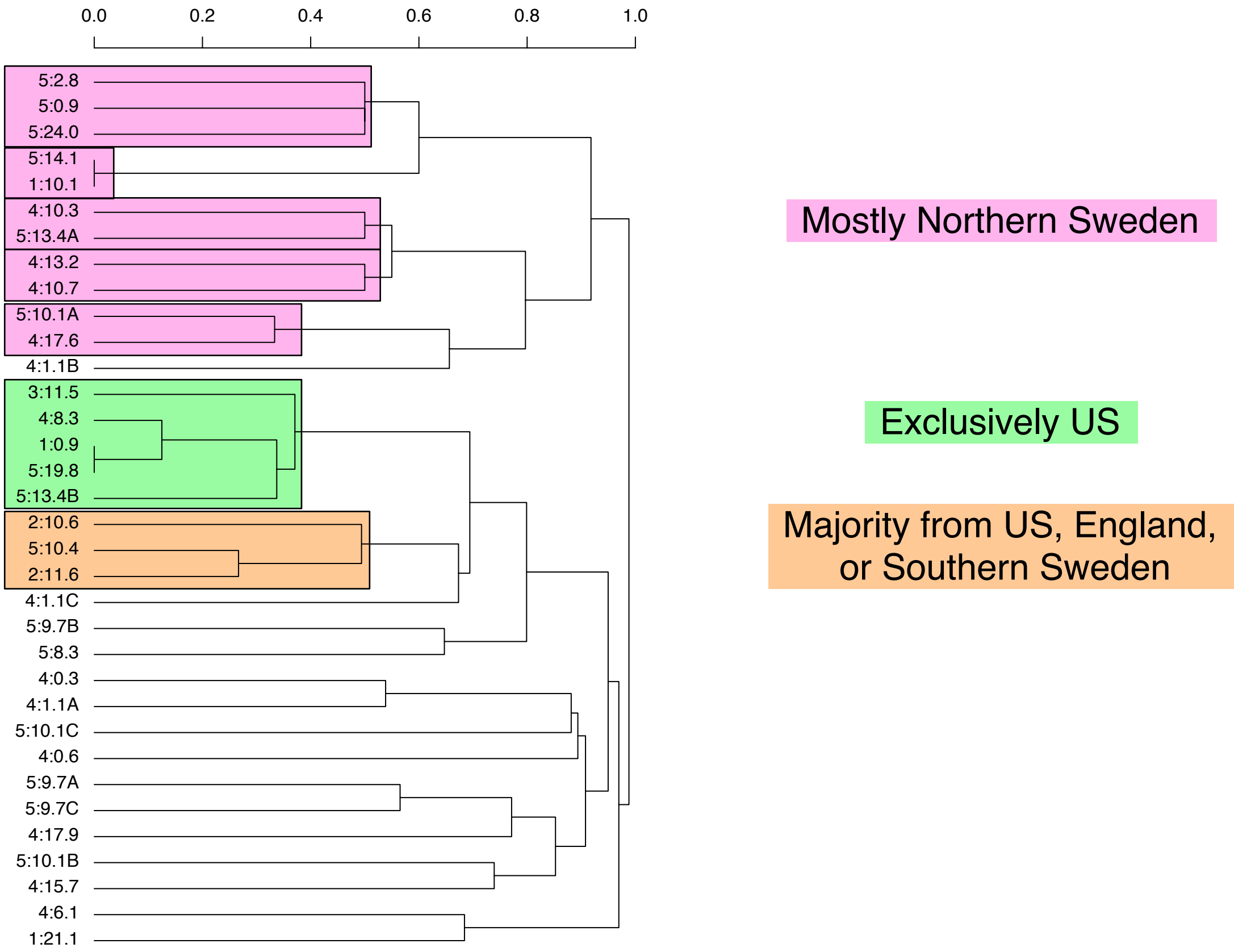
0.0
0.2
0.4
0.6
0.8
1.0
5:2.8
5:0.9
5:24.0
5:14.1
1:10.1
4:10.3
5:13.4A
4:13.2
4:10.7
5:10.1A
4:17.6
4:1.1B
3:11.5
4:8.3
1:0.9
5:19.8
5:13.4B
2:10.6
5:10.4
2:11.6
4:1.1C
5:9.7B
5:8.3
4:0.3
4:1.1A
5:10.1C
4:0.6
5:9.7A
5:9.7C
4:17.9
5:10.1B
4:15.7
4:6.1
1:21.1
Mostly Northern Sweden
Exclusively US
Majority from US, England, or Southern Sweden

EHH

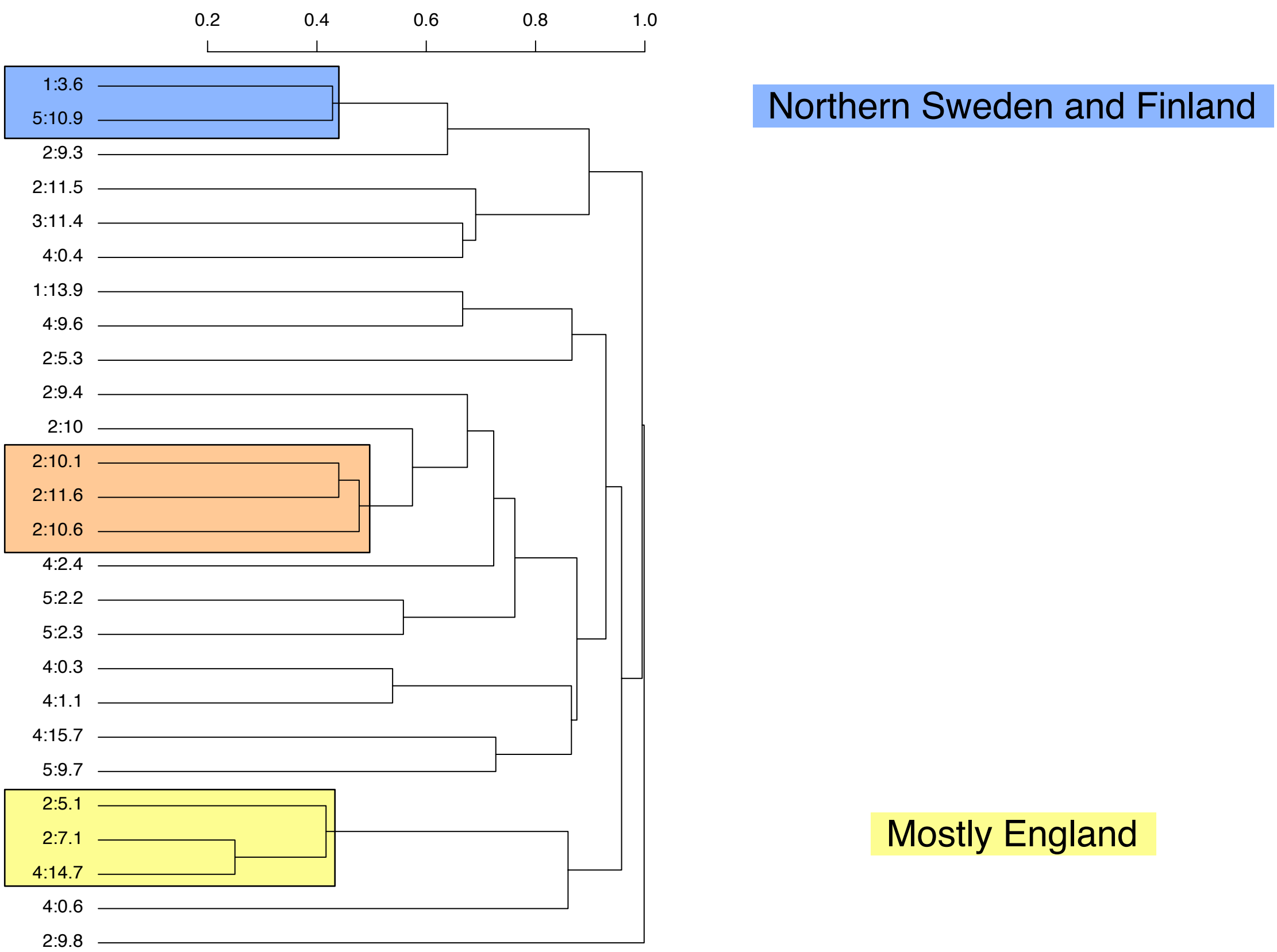
0.2
0.4
0.6
0.8
1.0
1:3.6
5:10.9
2:9.3
2:11.5
3:11.4
4:0.4
1:13.9
4:9.6
2:5.3
2:9.4
2:10
2:10.1
2:11.6
2:10.6
4:2.4
5:2.2
5:2.3
4:0.3
4:1.1
4:15.7
5:9.7
2:5.1
2:7.1
4:14.7
4:0.6
2:9.8
Northern Sweden and Finland
Mostly England

PHS
