# Supplementary material for: A Nonparametric Test Reveals Selection for Rapid Flowering in the Arabidopsis Genome
Source: PLoS Biol. 2006 Apr 25;4(5):e137. doi: 10.1371/journal.pbio.0040137 (PMC1440937; doi:10.1371/journal.pbio.0040137)
Supplement: Supplementary file 4 [file pbio.0040137.sg004.pdf]

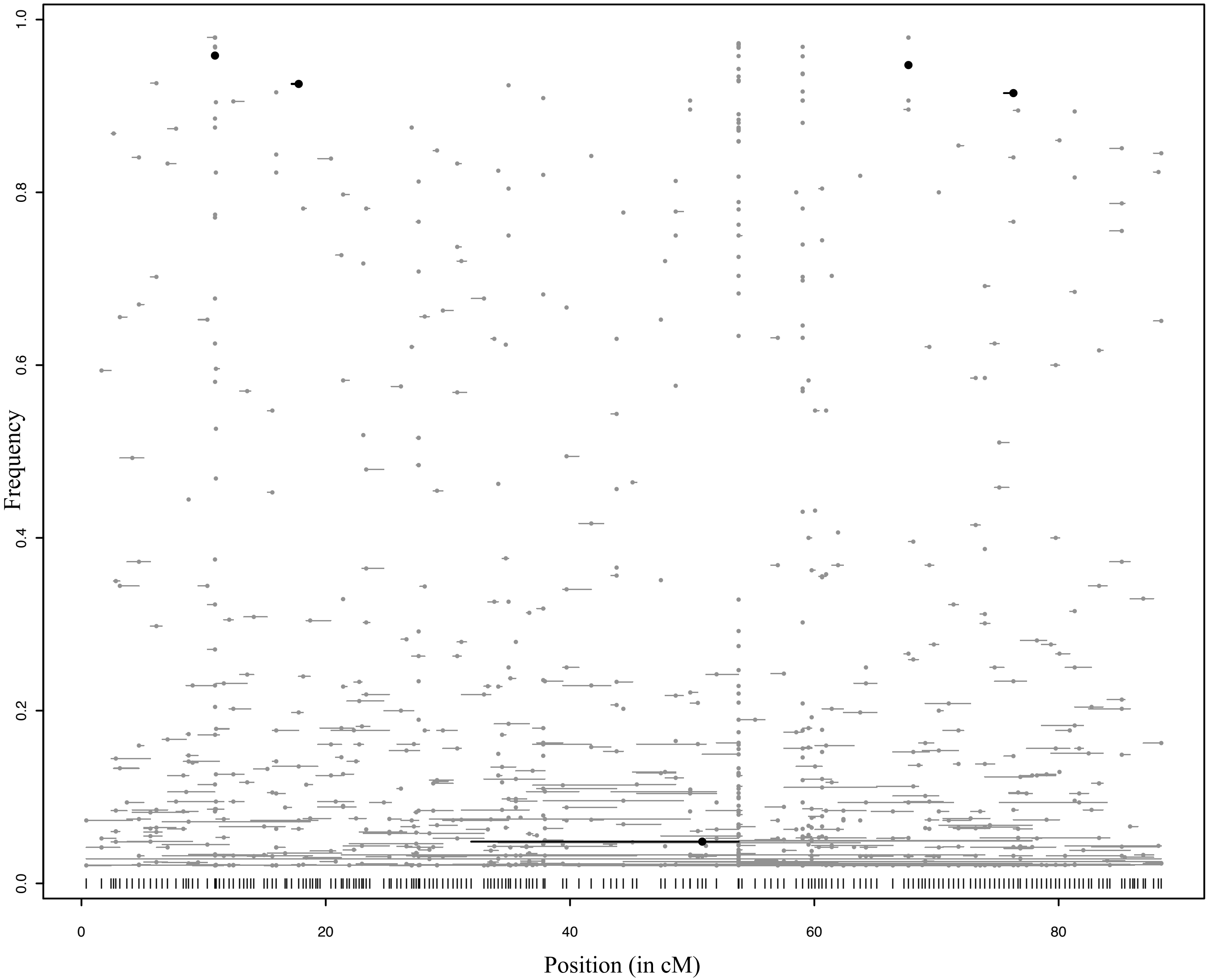

Frequency
1.0
0.8
0.6
0.4
0.2
0.0
0
20
40
60
80
Position (in cM)
