# Supplementary material for: A Nonparametric Test Reveals Selection for Rapid Flowering in the Arabidopsis Genome
Source: PLoS Biol. 2006 Apr 25;4(5):e137. doi: 10.1371/journal.pbio.0040137 (PMC1440937; doi:10.1371/journal.pbio.0040137)
Supplement: Supplementary file 10 [file pbio.0040137.sg011.pdf]

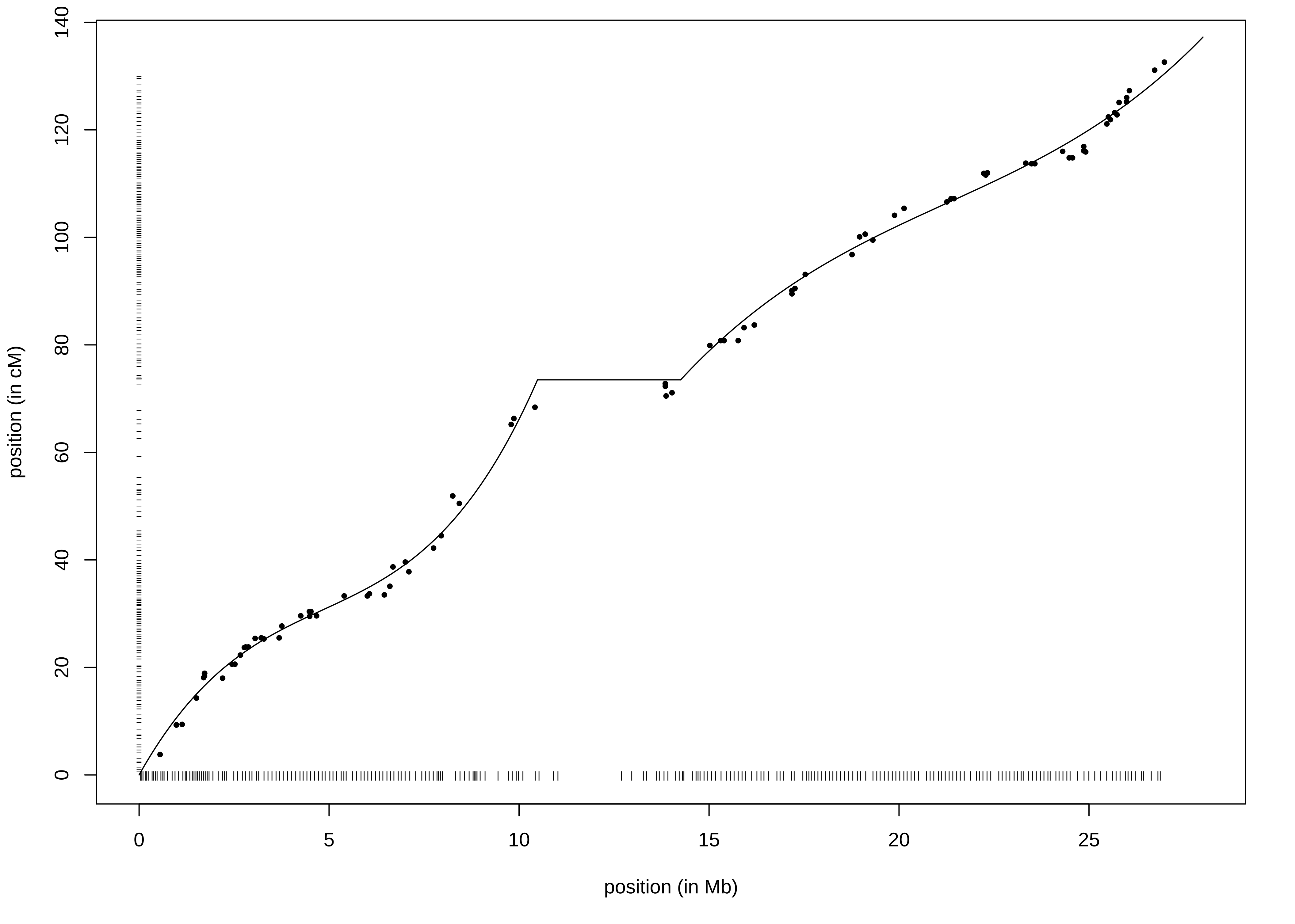
position (in cM)
0
20
40
60
80
100
120
140
0
5
10
15
20
25
position (in Mb)
